# Demographic History, Adaptation, and NRAP Convergent Evolution at Amino Acid Residue 100 in the World Northernmost Cattle from Siberia

**DOI:** 10.1093/molbev/msab078

**Published:** 2021-03-30

**Authors:** Laura Buggiotti, Andrey A Yurchenko, Nikolay S Yudin, Christy J Vander Jagt, Nadezhda V Vorobieva, Mariya A Kusliy, Sergei K Vasiliev, Andrey N Rodionov, Oksana I Boronetskaya, Natalia A Zinovieva, Alexander S Graphodatsky, Hans D Daetwyler, Denis M Larkin

**Affiliations:** 1 Royal Veterinary College, University of London, London, United Kingdom; 2 The Federal Research Center Institute of Cytology and Genetics, Siberian Branch of the Russian Academy of Sciences (ICG SB RAS), Novosibirsk, Russia; 3 Kurchatov Genomics Center, Institute of Cytology and Genetics, Siberian Branch of the Russian Academy of Science, Novosibirsk, Russia; 4 Agriculture Victoria, AgriBio, Centre for AgriBioscience, Bundoora, VIC, Australia; 5 Department of the Diversity and Evolution of Genomes, Institute of Molecular and Cellular Biology SB RAS, Novosibirsk, Russia; 6 Paleometal Archeology Department, Institute of Archaeology and Ethnography SB RAS, Novosibirsk, Russia; 7 L.K. Ernst Federal Research Centre for Animal Husbandry, Podolsk, Russia; 8 Moscow Agrarian Academy, Timiryazev Russian State Agrarian University, Moscow, Russia; 9 School of Applied Systems Biology, La Trobe University, Bundoora, VIC, Australia

**Keywords:** cold adaptation, Yakut cattle, convergent evolution, NRAP, Kholmogory cattle, resequencing

## Abstract

Native cattle breeds represent an important cultural heritage. They are a reservoir of genetic variation useful for properly responding to agriculture needs in the light of ongoing climate changes. Evolutionary processes that occur in response to extreme environmental conditions could also be better understood using adapted local populations. Herein, different evolutionary histories of the world northernmost native cattle breeds from Russia were investigated. They highlighted Kholmogory as a typical taurine cattle, whereas Yakut cattle separated from European taurines approximately 5,000 years ago and contain numerous ancestral and some novel genetic variants allowing their adaptation to harsh conditions of living above the Polar Circle. Scans for selection signatures pointed to several common gene pathways related to adaptation to harsh climates in both breeds. But genes affected by selection from these pathways were mostly different. A Yakut cattle breed-specific missense mutation in a highly conserved *NRAP* gene represents a unique example of a young amino acid residue convergent change shared with at least 16 species of hibernating/cold-adapted mammals from six distinct phylogenetic orders. This suggests a convergent evolution event along the mammalian phylogenetic tree and fast fixation in a single isolated cattle population exposed to a harsh climate.

## Introduction

Cattle domestication occurred approximately 8,000–10,000 years ago as a result of at least two independent events in the Fertile Crescent and the Indus Valley from two *Bos* subspecies, *Bos taurus* and *B. indicus* (Loftus et al. 1994; Larkin and Yudin 2016). Many breeds adapted to a vast variety of environmental conditions originate from interbreeding between the *B. taurus* (taurine) and *B. indicus* (indicine) populations (Larkin and Yudin 2016) and admixture with several other Bovinae species, including yak, banteng, and gaur (Decker et al. 2014; Medugorac et al. 2017). Although adaptations of cattle breeds to hot climates are relatively well studied due to the economic needs in the light of adaptation to hot, tropical environments (Renaudeau et al. 2012; Bernabucci et al. 2014; Sinha et al. 2017), there is limited knowledge about the mechanisms of cattle adaptation to colder climates with most studies limited to genotyping data sets (Igoshin et al. 2019) rather than whole-genome (re)sequencing. There are several northern cattle breeds that are of a particular interest for such studies. Two of them originate from, and are adapted to, the harsh climates of Russia: the Kholmogory and the Yakut cattle. The Kholmogory cattle was formed in the European part of Russia, about 300 years ago, from local taurine landraces which were crossed with “Dutch cattle” in the 18th century (Felius et al. 2014). The Yakut cattle were likely formed at the Baikal lake area of Siberia (Crate 2006) and migrated together with Yakut people to Yakutia region about 800 years ago (Crate 2006). They now are the world northernmost cattle which can be found up to 200 km above the Polar Circle, where they are exposed to long winters and low temperatures that can drop to below −70 °C. They were likely influenced by Asian cattle populations and belong therefore to the “Turano–Mongolian” cattle group. They are the only extant pure and commercial Turano–Mongolian population as most other breeds were interbred with European taurines to improve performance (Felius et al. 2014). Indeed, a recent phylogenetic analysis placed the Yakut cattle together with other Turano–Mongolian breeds, including the Hanwoo and Japanese Black, but at the same time suggested possible historical admixture with indicine and African taurine cattle (Yurchenko, Yudin, et al. 2018). However, the origin of the Turano–Mongolian cattle group remains unclear with some authors suggesting an independent domestication event in Eastern Asia (Mannen et al. 2004; Li et al. 2007).

In this study, the Yakut and Kholmogory cattle were compared with their phylogenetically closest breeds to identify signatures of selection that could have influence on their adaptations. The demographic and admixture analyses of the two populations suggest that the Kholmogory and Yakut cattle became separated about 4,000–5,000 years ago. Our data imply that the Yakut cattle genome contains a distinct fraction of ancestral cattle variants not found in the European taurines. The two breeds demonstrate signatures of selection in the same gene pathways related to adaptation to harsh climates, but the genes affected by selection from these pathways are mostly different. All Yakut cattle individuals contain a missense mutation in a highly conserved *NRAP* gene involved in the heart function. This change is shared with a majority of hibernating mammals but absent from all other cattle breeds, ancient DNA samples from the genus *Bos* and a majority of mammalian species, suggesting a recent convergent evolution event and fast selection in a single isolated cattle population exposed to a harsh climate.

## Results

### Whole-Genome Resequencing, Mapping, and SNP Detection

Resequencing of the Yakut and Kholmogory cattle breeds (40 samples; 20 per breed) resulted in a total of 1,667.6 Gb of filtered sequence data. The read mapping rate against the reference genome for Kholmogory cattle was 99.6%, whereas for the Yakut cattle, it was 99.5%, resulting in an average sequencing coverage of 11.7× and 11.2× for the Kholmogory and Yakut samples, respectively (table 1). The total number of SNPs was 17.0 and 15.4 million for the Yakut and Kholmogory samples, respectively. To identify signatures of selection differentiating the Yakut and Kholmogory cattle breeds from phylogenetically most related breeds (Yurchenko, Yudin, et al. 2018), 20 samples of Holstein and 20 samples of Hanwoo breeds were included in our data set. The average sequencing coverage per sample was 10.6× for Holstein and 10.2× for Hanwoo. A total of 31 million SNPs were called in the four-breed joint set of which 27.5 million passed our quality filtering criteria. As expected, the majority (21.7 million [78.9%]) of the SNPs was located in the intergenic and intronic regions. About 0.6%, 0.3%, and 0.6% of the SNPs overlapped mammalian, cetartiodactyl, and ruminant conserved noncoding elements (Farré et al. 2019) and the number of synonymous and missense SNPs were 109,774 and 64,221, respectively (table 1). There were 35,919 and 31,089 missense variants in the Russian Yakut and Kholmogory breeds, respectively. We found 224 and 210 nonsense variants in the Yakut and Kholmogory breeds, of which 32.6% and 21.9% were found in a single breed, respectively. A total of 678 and 594 SNPs were classified as “essential splice variants” whereas 17 and 15 were stop losses, in the Yakut and Kholmogory breeds, respectively (supplementary information 1, Supplementary Material online).

**Table 1.** Summary of Samples and Genome Sequencing Statistics (hard filtered biallelic SNPs).

	Holstein	Kholmogory	Hanwoo	Yakut	Total
Samples	20	20	20	20	80
Coverage	10.60 ± 5.1	11.68 ± 1.2	10.21 ± 3.13	11.18 ± 0.76	
SNPs	14,448,782	15,426,893	18,914,454	17,025,030	27,487,145
ts/tv	2.13	2.20	2.23	2.23	2.15
Synonymous^a^	56,249	49,976	56,568	75,798	109,774
Missense^a^	32,216	31,089	35,740	35,919	64,221
CDS	53,523	63,551	70,590	92,202	137,744
3′ UTR	33,537	36,100	45,514	46,886	74,332
5′ UTR	6,345	7,419	8,473	8,557	13,814
Intron	2,927,686	3,100,776	3,898,489	3,457,265	5,676,380
Intergenic	11,489,562	12,280,607	14,965,760	13,485,886	21,685,165
MamCNEs	82,533	85,465	108,525	89,593	167,632
CetCNEs	42,643	43,811	56,327	45,934	84,970
RumCNEs	74,576	76,886	100,8716	83,653	153,684

Note.—CNEs, conserved noncoding elements; MamCNEs, mammalian CNEs; CetCNEs, cetartiodactyl CNEs; RumCNEs, ruminant CNEs.

a Synonymous and missense mutations were annotated by dNdScv.

To identify historical relationships and admixture between the Yakut cattle and other cattle breeds and species with potential historical admixture, SNPs from additional 113 individuals, representing indicine (African, Chinese, and Indian) cattle and five Bovinae species (yak, banteng, gayal, gaur, bison), were combined with the four breed SNP sets, resulting in a total of 193 individuals (supplementary information 2, Supplementary Material online) and 11 million high-quality biallelic SNPs.

### Demographic History and Population Divergence

**Fig. 1.**
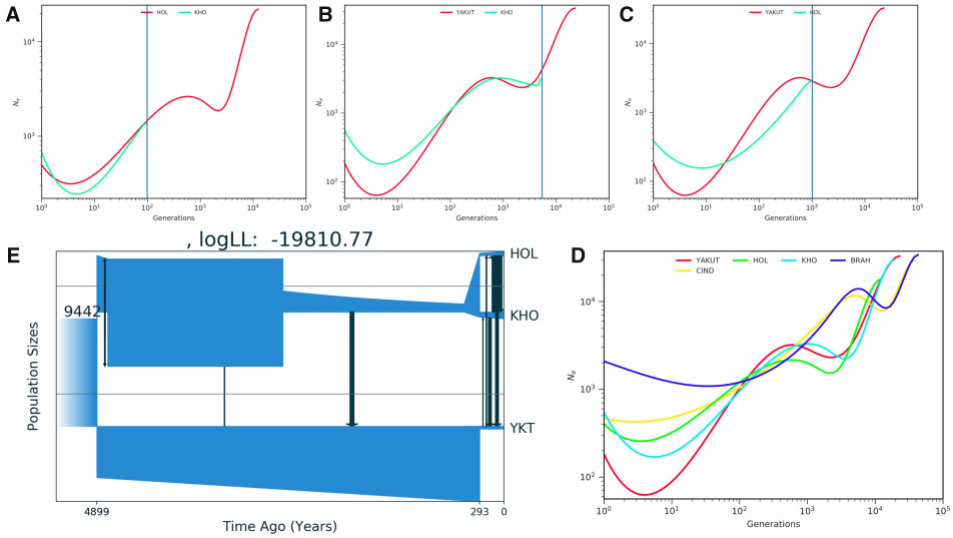
Demographic history of Yakut and Kholmogory cattle breeds. SMC++-inferred effective population sizes (Ne) with respect to time (generations) for (*A*) Holstein (HOL) and Kholmogory (KHO), (*B*) Yakut and KHO, (*C*) Yakut and HOL, and (*D*) Yakut, HOL, KHO, BRAH (Brahman), and CIND (Chinese indicine). Blue lines highlight separation time. (*E*) Demographic model for Holstein, Kholmogory, and Yakut cattle breeds using GADMA.

We estimated demographic history using two distinct approaches: coalescent-based SMC++ (Terhorst et al. 2017) and ordinary differential equations (moments) realized in the GADMA package (Noskova et al. 2020), applied to our resequencing set (table 1; supplementary information 2, Supplementary Material online), closely related breeds (Holstein for Kholmogory and Hanwoo for Yakut cattle) and indicine cattle. SMC++ algorithm estimated a stepwise decline in the population size of cattle breeds with indicine lineage (Brahman) exhibiting the most divergent and a much less pronounced recent decline. Yakut cattle demonstrated much stronger population decline more recently (around 200–500 years ago) than Kholmogory and Holstein (fig. 1*D*). The divergence time of Holstein and Kholmogory was estimated to be around 500 years (fig. 1*A*), whereas the divergence of Yakut lineage was much earlier with a 5,000-year estimation for Yakut–Holstein divergence (fig. 1*C*) and 25,000 years for Yakut-Kholmogory divergence (fig. 1*B*). The later estimation is likely to be an overestimation (i.e., predomestication) because the SMC++ does not consider any possible migration events between populations.

To understand demographic histories of the Kholmogory and Yakut cattle breeds we focused to the three breeds with largest sample data. GADMA returned a quite realistic demographic population model of Yakut, Kholmogory, and Holstein divergence (fig. 1*E*). The estimated divergence between Kholmogory and Holstein was around 300 years ago in line with historical data, whereas the divergence of Yakut cattle from the common ancestor of Holstein and Kholmogory was estimated to be around 4,900 years ago. The best Log-likelihood model also indicated sharp decline of Yakut cattle population around 300 years ago and migrational events from Holstein, Kholmogory and their common ancestor to the Yakut lineage (fig. 1*E*). Thus, the very different methods with contrasting assumptions (allowed and not migrational events) point out to a very old (several thousands of years) time of divergence of the Yakut breed from the common ancestor of modern taurine breeds, which was accompanied also by a historically very recent bottleneck of Yakut cattle population.

### Yakut Cattle: Indicine Introgression or Ancestral Taurine Genetic Variants

**Fig. 2.**
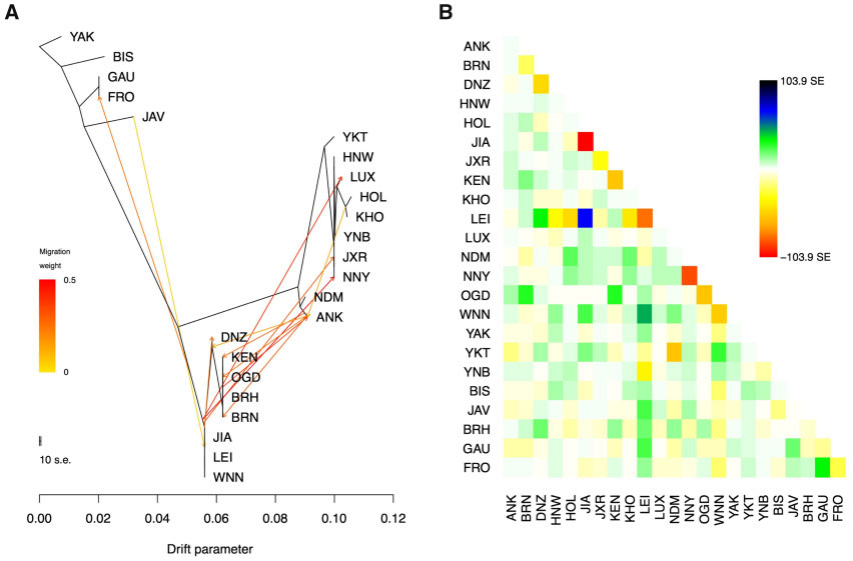
(*A*) The maximum-likelihood tree generated by TreeMix (up to the migration 12). The scale bar shows ten times the average standard error of the entries in the sample covariance matrix. Abbreviations of the populations are as follow: BIS, Bison; GAU, Gaur; FRO, Gayal; JAV, Banteng; YKT, Yakut; HNW, Hanwoo; LUX, Luxi; HOL, Holstein; KHO, Kholmogory; YNB, Yanbian; JXR, Jiaxian Red; NNY, Nanyang; NDM, N’Dama; ANK, Ankole; DNZ, Dianzhong; KEN, Kenana; OGD, Ogaden; BRH, Brahman; BRN, Boran; JIA, Ji'an; LEI, Leiqiong; WNN, Wannan. (*B*) Residuals of the maximum-likelihood phylogenetic tree. As implemented in TreeMix, the residual covariance between each pair of populations i and j is divided by the average standard error across all pairs. This scaled residual is then plotted in each cell (i, j). Colors are described in the palette on the right. Residuals above zero represent populations that are more closely related to each other in the data than in the best-fit tree and thus are candidates for admixture events (SE, standard error).

The Yakut cattle were likely formed in Asia and demonstrated signatures of possible introgression from indicine cattle (Yurchenko, Yudin, et al. 2018). To identify introgressed genome intervals, a combination of several approaches has been used in this study. We used “pure” taurine (Holstein, Kholmogory) and indicine (African, Chinese, and Indian) breeds as reference populations in the RFMix analysis, which suggested that the Yakut cattle contain approximately 86.5% of the “taurine-like” and approximately 13.5% of the “indicine-like” genome. The TreeMix analysis, using a total of 18 cattle breeds and five Bovinae species did not detect any significant introgression between the Yakut cattle and other populations used (fig. 2). On the TreeMix tree the Yakut cattle was placed close to the Hanwoo and Yanbian breeds and the Kholmogory close to Holstein, which agrees with previous studies (Yurchenko, Daetwyler, et al. 2018; Yurchenko, Yudin, et al. 2018). The *f3* statistics calculated on population triples using the Yakut cattle as a target, various indicine cattle breeds and five related species as source populations did not result in a significant Z-score (supplementary information 3a, Supplementary Material online). The *D* statistics (Bovinae species, indicine, Yakut, Yak) results, however, suggested admixture between the Yakut and indicine cattle as well as with taurine breeds (supplementary information 3b and supplementary fig. 1, Supplementary Material online).

**Fig. 3.**
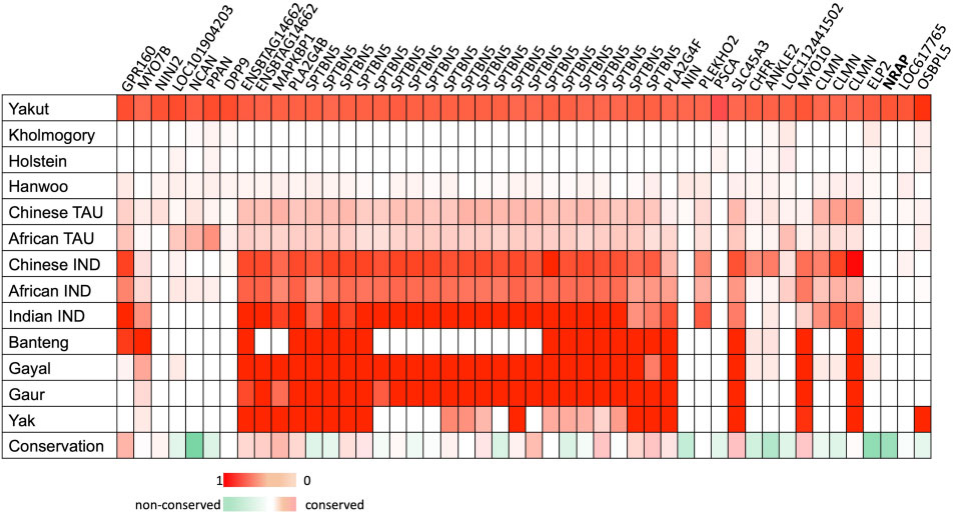
Forty eight missense SNPs with high frequency alleles present in Yakut cattle (≥70%) but with low frequency of the same allele in Holstein, Kholmogory, and Hanwoo (≤10%); shades of red represent allele frequency and conservation score for Yakut cattle allele encoded amino acids more often found in orthologous positions of other mammals, whereas shades of green indicate those Yakut cattle allele encoded amino acids that are found rarely in other species; TAU, taurine; IND, indicine.

To investigate reasons for these conflicting results we extracted four sets of biallelic SNPs with high derived allele frequencies in the Yakut, Kholmogory, Holstein, and Hanwoo genomes but with low frequency of the same allele in the other three breeds (one breed MAF ≥ 0.7; other three breeds MAF ≤ 0.1; autosomal). The number of such SNPs was 13,257 in the Yakut cattle but much lower in the other breeds: 758 for Hanwoo, 103 for Kholmogory, and 311 for Holstein. Of the 13,257 Yakut SNPs, the majority (9,809 [73.92%]) overlapped the Yakut cattle genome intervals identified as “indicine-like” by the RFMix analysis. To look into the evolutionary history of the high-frequency Yakut alleles, we focused on all 48 missense mutations present in the 13,257 SNP set (table 2; fig. 3). Of these, 37 Yakut cattle high-frequency derived alleles were absent from the European taurine cattle (Holsten and Kholmogory), but most of them had high frequencies in the indicine cattle breeds. Furthermore, 30/37 derived alleles were found in at least one additional Bovinae species suggesting that they could be present in the ancestral taurine genome and could be eliminated from at least two European taurine breed (Holstein and Kholmogory) genomes due to selection or drift. To check this hypothesis, we looked into the orthologous positions in proteins of up to 82 evolutionary distinct animals (ranging from mammals to fish), and found that for 10/30 positions, the Hereford reference (*Btau6*) amino acids matched the amino acid preferably found in the orthologous position of other animals, whereas for the 19/30 positions, the Yakut cattle amino acid was overrepresented in other animal proteins. These observations coupled with the TreeMix and *f3* statistics results support the hypothesis that most of the high-frequency Yakut cattle SNPs could represent alleles eliminated from the European taurine cattle. For the final check, we looked for the presence of these 48 missense SNPs in the non-European taurine breeds and found 37/48 Yakut cattle alleles in Hanwoo, 46/48 in the Chinese, and 42/48 in the African (N’Dama) taurine breeds confirming our hypothesis that these variants could be present in the ancestral taurine genome.

**Table 2.** High-Frequency Derived Alleles in Yakut Cattle Genomes.

SNPs	Statistics
High-frequency allele in Yakut cattle genomes^a^	13,257
Yakut cattle-specific	263
Low frequency in European taurine genomes^b^	4,866
Absent from European taurine genomes	8,128
Found in indicine genomes	7,546
Found in Bovinae genomes	5,760
Found *only* in African, Chinese, and Asian taurine genomes	2,018
Found *only* in Hanwoo genomes	184
Found *only* in indicine genomes	166
Missense alleles	48
Found in Chinese taurine genomes	46
Found in African taurine genomes	42
Found in indicine genomes	39
Found in Hanwoo genomes	37
Absent from European taurine genomes	37
Found in Bovinae genomes	30
Evolutionary conserved (Yakut cattle allele)	19
Evolutionary conserved (European taurine allele)	10
Yakut cattle-specific	1

a Allele frequency ≥70% in Yakut cattle genomes and <10% in Hanwoo, Holstein, and Kholmogory cattle genomes.

b <10% in Holstein, and Kholmogory cattle genomes.

We detected only one Yakut cattle missense derived allele not found in the indicine, taurine cattle, or other Bovinae species, in the NRAP protein. The fact that all 48 SNPs represent alleles different from the reference genome (Hereford) suggests that they are likely to be absent from both the dairy and beef European taurines. Indeed, we found only 331 SNPs for which the Yakut cattle had a high-frequency allele identical to the reference Hereford genome, whereas Kholmogory and Holstein had high-frequency derived alleles. None of these 331 SNPs were missense mutations.

### Putative Ancestral Taurine Alleles Absent or Nearly Absent in the European Taurine Breeds

**Fig. 4.**
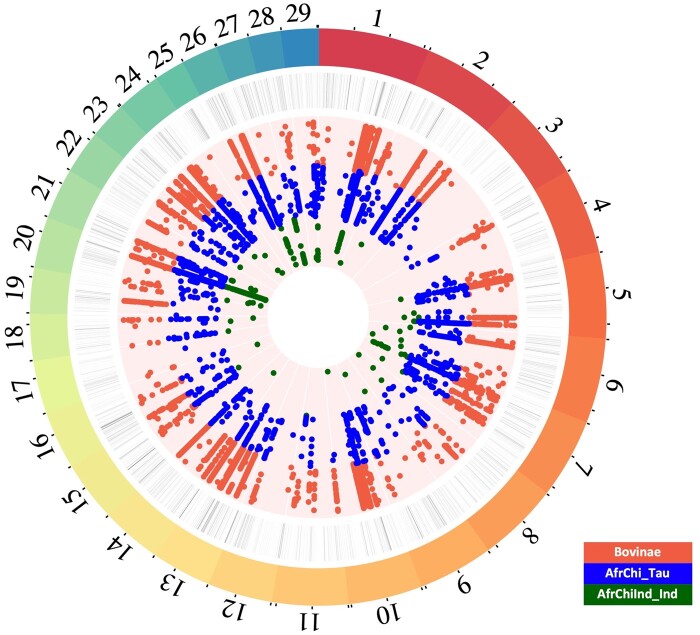
High frequency SNPs in Yakut cattle but low frequency/absence of the same allele in Holstein, Kholmogory, and Hanwoo (total of 13,257 SNP). Each circle from the periphery to the center shows the following data sets: bovine autosomes, indicine-like genome intervals from the RFMix analysis in different shades of gray (light to dark indicating indicine-like interval frequencies in the Yakut population ranging from 50% to 100%), Yakut alleles found in Bovinae species in red, in African and Chinese taurine breeds in blue, and exclusively in Indian, African, and Chinese indicine breeds in green.

We used the whole set of 13,257 high-frequency Yakut cattle SNPs to identify putative regions of the ancestral taurine genome with haplotypes that are present in high frequency in the Yakut cattle but are absent/nearly absent from the European taurines. Among the 13,257 SNPs, we found 263 alleles present in the Yakut cattle only, 4,866 alleles found in a low frequency (<10%) in the European taurines and 8,128 alleles absent from the European taurine set. Of the 8,128 SNPs, 7,546 (92.83%) were found in indicine breeds; however, only 166 (2.00%) of them were indicine-specific, whereas 5,760 (70.87%) were also present in at least one additional Bovinae genome, 2,018 (24.82%) additional SNPs were present in either the African or the Chinese taurines, and 184 (2.26%) were shared with Hanwoo only (table 2; fig. 4 and supplementary fig. 2, Supplementary Material online). These results confirm that the “indicine-like” genome regions of the Yakut cattle to a large extent, if not all, represent ancestral taurine haplotypes mostly eliminated or present in a low frequency in the European taurine genomes. As a final test, we identified all intervals in the Yakut cattle genome containing 1.67 million “indicine-like” alleles as assigned by the RFMix analysis, intersected them with Bovinae, African, Chinese taurine genomes, and Hanwoo, then eliminated all intersected intervals except for those which contained indicine-specific alleles (155,542 SNPs) and repeated the RFMix analysis. It showed that the filtered set had 2.5% of the “indicine-like” genome suggesting that the removed intervals were responsible for the majority of the “indicine-like” part of the Yakut cattle genome.

**Fig. 5.**
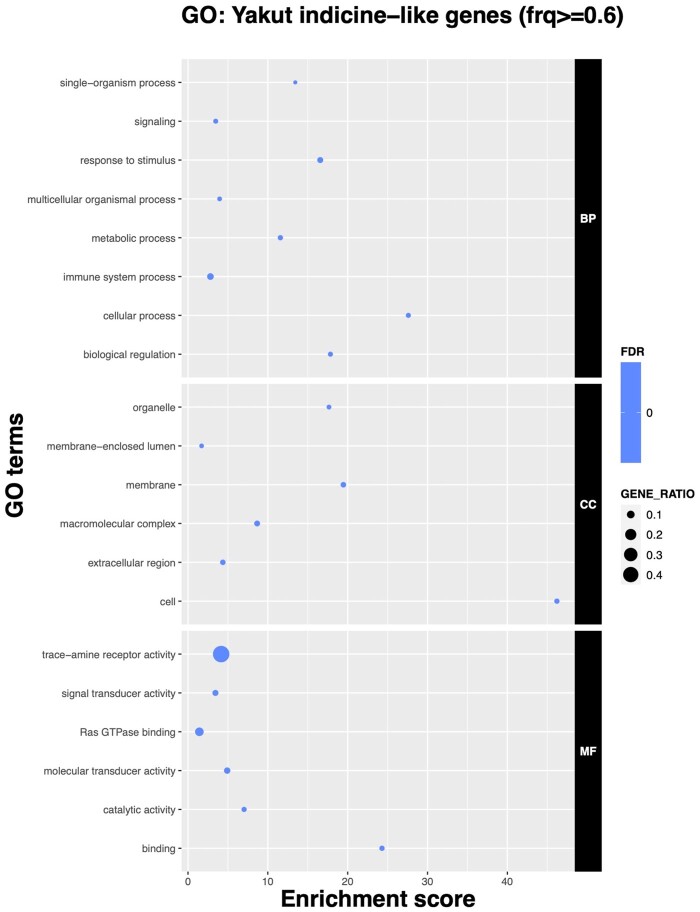
GO analysis of genes overlapping “indicine-like” genome intervals (≥60% of the total gene length) as identified by RFMix.

We extracted all genes found in the ancestral and European taurine genome intervals (gene total length overlap ≥60% for a gene to be assigned to an either set). A total of 1,639 (6.7%) genes were assigned to the ancestral and 19,205 (78.2%) genes to the European taurine segments. GO enrichment analysis of the genes in ancestral segments revealed the term “*response to stimulus*” (*q*-value < 0.02) and “*metabolic process*” (*q*-value < 0.02) among others (fig. 5; supplementary information 4a, Supplementary Material online). Moreover, the genes from the ancestral segments were enriched in the *“immune system process”* pathway (*q*-value < 0.02; fig. 5) including the MHC class II antigens. The top DAVID cluster (enrichment score 3.68) included multiple MHC class II antigens, immune genes (e.g., *IL36A* and *IL36B*), heat shock proteins (*HSPA1A* and *HSPA1L*), and the insulin receptor (*INSR*; supplementary information 4b, Supplementary Material online). The GO category *“response to pain”* was significantly enriched in genes found in ancestral segments identified by RFMix (l2-fold = 1.21, *P*-value = 5.00e-03, *q*-value = 1.00e-02; 10,000 permutation test; supplementary information 5, Supplementary Material online).

We then looked closer into the 25 genes that had high-frequency derived missense alleles in the Yakut genomes (fig. 3). A gene with the largest number of such variants was the actin-binding *SPTBN5* containing 21 missense mutations. Not surprisingly, the region containing the *SPTBN5* also demonstrated the highest F_ST_ value between the Yakut cattle and all taurine breeds from the Run 7 of the 1000 Bull Genome project. When all breeds of the Run 7 were contrasted against the Yakut cattle, *SPTBN5* was still on top of the list (top fourth gene position, with the BOLA-DQB MHC class II antigen being the number one; supplementary information 6, Supplementary Material online). We found high-frequency derived alleles in other actin-binding genes, including the *MYO10*, *CLMN*, and the *NRAP*. Among these, the *MYO10* also contained a unique missense mutation in the Yakut horse (Librado et al. 2015), suggesting its probable contribution to the local adaptation of distinct domesticated species to harsh Siberian climates.

### Yakut Cattle-specific NRAP Missense Mutation

The *NRAP* variant was the only Yakut-cattle specific missense mutation which was not observed in other cattle breeds nor the Bovinae species in our set. Out of 19 Yakut cattle individuals with high coverage in this region (no. reads >5), 12 were homozygous for the derived allele whereas seven were heterozygous. We checked this position in the complete 1000 Bull Genome Run 7 data set (3,767 individuals, indicine and taurine breeds). The frequency of the reference (G) allele was 0.995, whereas the frequency of the derived (T) allele was 0.005. The only four additional (to our Yakut cattle samples) animals which contained the same derived allele were four Yakut cattle samples obtained from the NCBI SRA archive, suggesting that this genetic variant is Yakut cattle-specific.

**Fig. 6.**
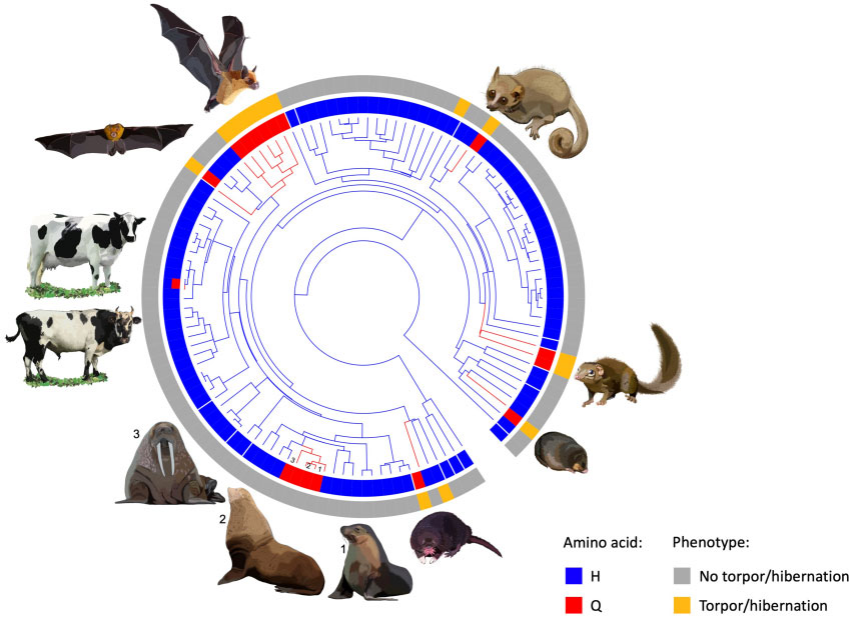
Phylogenetic tree of 114 mammals from nine phylogenetic orders (Kumar et al. 2017). Carriers of the common histidine (H) amino acid are in blue, whereas the carries of the glutamine (Q) variant are in red (cattle has both blue and red indicating the Yakut cattle specific H100Q mutation); the outer circle in orange highlights species that either hibernate or enter torpor (supplementary information 15, Supplementary Material online). Images are representative of the species that have the glutamine variant, Yakut cattle (glutamine/histidine) and Kholmogory cattle (histidine).

**Fig. 7.**
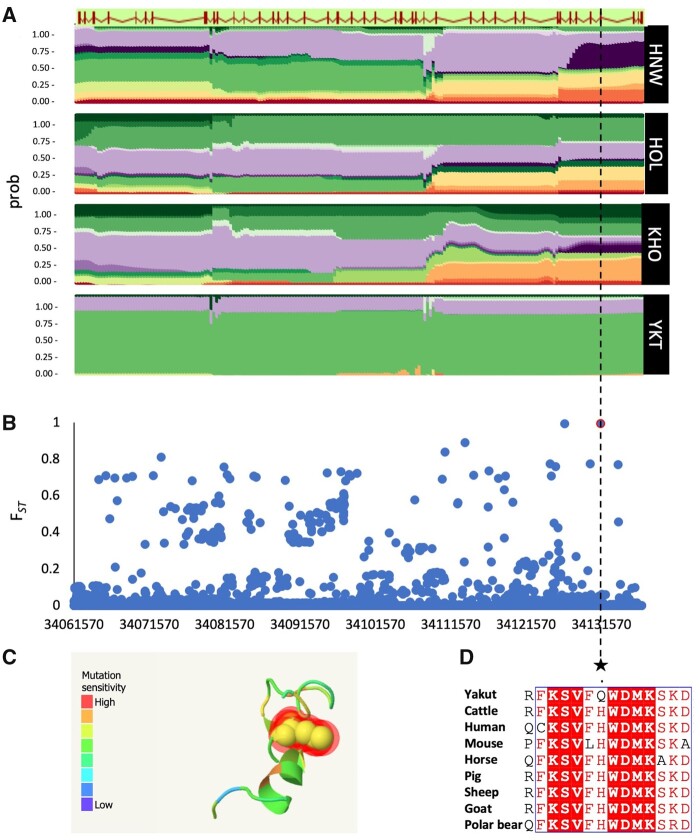
SNPs in the *NRAP* gene. (*A*) Haplotype diversity in the *NRAP* gene for Hanwoo (HNW), Holstein (HOL), Kholmogory (KHO), and Yakut (YKT) breeds. (*B*) *F*_ST_ analysis of SNPs from the 1000 Bull Genomes Run 7 in the Yakut population against all other individuals; (*C*) Sensitivity of the H100Q change modeled in the NRAP protein; (*D*) Extract of local alignment of the exon 4 of the NRAP protein showing the H100Q mutation and adjacent amino acids in the Yakut cattle (Yakut), reference Hereford genome (Cattle), and seven mammals.

The nebulin-related-anchoring protein (NRAP) is a highly evolutionary conserved actin-binding cytoskeletal protein expressed exclusively in the skeletal and cardiac muscles, and is involved in myofibrillar assembly and force transmission in the heart (Truszkowska et al. 2017). Our phylogenetic comparison shows that the H100Q variant observed in the Yakut cattle is rare in other mammals (found in 16/113 species), but is preferably present in species that either hibernate, for example, little brown bat and other hibernating bats and mouse lemur or enter seasonal torpor, for example, cape golden mole and tree shrew. Our statistical analysis using phylogenetic logistic regression (Tung Ho and Ané 2014) considering phylogeny of the 113 species from nine mammalian orders (Hecker and Hiller 2020) shows phylogeny-independent significant correlation (*R*^2^ = 0.77, *P*-value = 2.72e-06, α = 0.00011) between the presence of the glutamine (Gln, Q) variant and cold adaptation, hibernation or ability to enter torpor (figs. 6 and 7, table 3). The four species that do not hibernate or enter torpor but still have the glutamine variant are the walrus, sea lion and two seals. Interestingly, walrus, seals and sea lions are capable of slowing heart rate to several beat per minute when diving (Williams et al. 2015; McKnight et al. 2019), whereas walrus is also cold-adapted suggesting that the same adaptation as in the Yakut cattle could be found in other large animals.

**Table 3.** Association between Allelic Variants at NRAP Residue 100 and Habitat across 113 Mammalian Species.

		No. Species			
Residue	Amino Acid	Torpor/Hibernation	No Torpor/Hibernation	*R* ^2^	*P-*value^a^
100	*His*	2^b^	95	0.77	2.72 × 10^−06^
	*Gln*	12	4		

a Phylogenetic logistic regression test.

b Tenrec hibernates when it is hot rather than cold and the alpine squirrel. One species, the golden hamster, has not been included in the analysis as the only hibernating species which shows arginine (Arg), a descendant of glutamine (Gln) at the NRAP 100 position.

To investigate possibilities of historical introgression of the *NRAP* mutation into the Yakut cattle genome from other *Bos* species, we performed search for this variant in 22 ancient DNA samples from Siberia (age range: 2,500–50,000 years; supplementary information 7, Supplementary Material online; 12 samples from *B. primigenius*, three from *B. taurus*, two from *B. mutus*, and five from *Bison priscus*). Resequencing of these samples did not identify the missense G > T change in the position chr26: 34,131,393, observed in the Yakut cattle. In addition, we checked two historical samples: one of the Yakut cattle (94 years old) and one of the extinct Siberian cattle breed (>100 years old). We identified the G > T change in the Yakut sample only. These results confirm that the H100Q change is indeed Yakut cattle-specific and is unlikely to originate from a historical or recent introgression into their genome.

There are multiple independent lines of evidence suggesting that the highly evolutionarily conserved NRAP protein could contribute to climate adaptations in various animal groups. For instance, in the Australian bearded dragon, *NRAP* is differentially expressed in the skeletal muscle and heart before and during hibernation (Capraro et al. 2019), whereas in the alligator *NRAP* expression was downregulated in the heart in response to hypoxia (Alderman et al. 2019). In the American eel, fixation of an alternative *NRAP* allele was associated with latitude and river water temperature in a landscape genomics study (Gagnaire et al. 2012). Interestingly, in a recent environmental GWAS study (Rowan et al. 2020), variants in the *NRAP* gene were associated with environmental adaptations in the US Simmental cattle.

In mice, *NRAP* is downregulated during dilated cardiomyopathy (DCM) (Ehler et al. 2001); human patients with DCM are homozygous for *NRAP* mutations (Truszkowska et al. 2017). Interestingly, in humans, the homologous *NRAP* position on HSA10:113,657,530 (GRCh38) contains a three-allelic (G/A/T) SNP (rs1891762) with a rare T allele leading to the H100Q change (allele frequency: 0.000007 [gnomAD v. 2.1.1]) and A (0.8955) > G (0.1045) change being synonymous. During DCM, pumping ability of the left ventricle of the heart is affected leading to issues with pumping blood out of the heart (Schultheiss et al. 2019). The human data suggest that under the normal conditions, the H100Q change is likely to be disadvantageous explaining why the T allele is found in the cold-adapted/hibernating/deep diving species only.

### Genomic Regions under Selection in World Northernmost Cattle Breeds

We identified 286 regions under selection in the Yakut and 219 in the Kholmogory cattle breeds using the HapFLK analyses (*q*-value < 0.01; supplementary information 8, Supplementary Material online), four and three genes under positive and 246 and 62 genes under negative selection using the d*N*/d*S* in the Yakut and Kholmogory breeds, respectively, when focusing on SNPs with a likely effect on the whole breed (applying the 60% MAF filter; supplementary information 9b, Supplementary Material online). There were 14 and four genes in the Yakut and Kholmogory cattle, respectively, from the HapFLK selected regions which were also picked up by the d*N*/d*S* analysis. Of these genes, *SPTBN5* was also found in the top 1% *F*_ST_ intervals in the Yakut cattle, whereas *HID1* (involved in regulation of neuropeptide sorting) in the Kholmogory cattle.

**Fig. 8.**
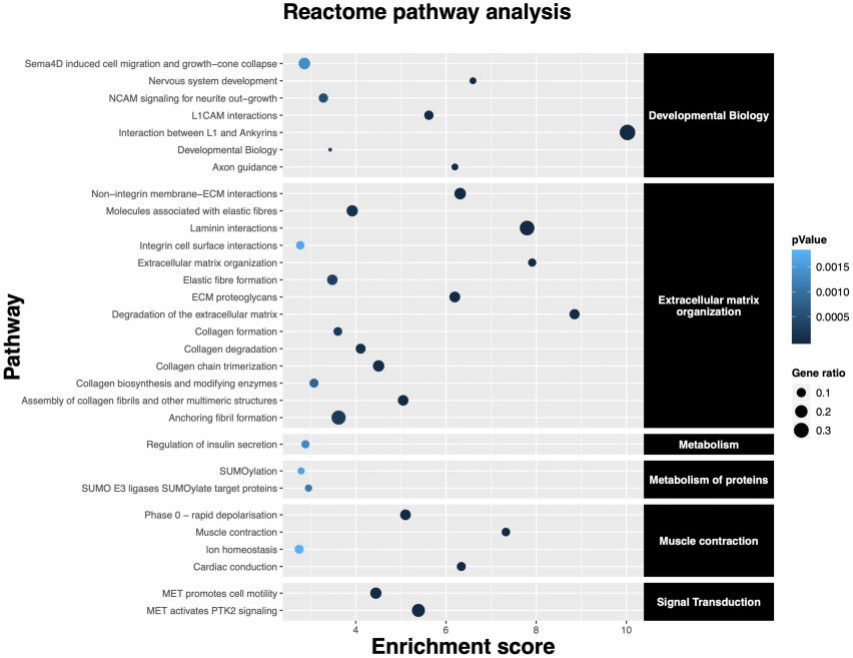
Reactome pathway enrichment analysis for Yakut cattle genes identified to be under selection by the d*N*/d*S* analysis (FDR < 0.05).

As previously shown, adaptation to harsh environments requires proper responses to external stimuli (Witter et al. 2012). Both the Yakut and Kholmogory breeds demonstrated significant enrichment for the *response to stimulus* GO category in the HapFLK regions, supported further by the d*N*/d*S* analysis of the Yakut cattle genes. Interestingly, in the Yakut cattle, this GO term was significantly overrepresented in genes containing high-frequency SNPs absent from the European taurines, suggesting that the genetic profile of selection between the two breeds is different. One of the strongest HapFLK signals in the Yakut cattle was observed for the gene *ZNF622*, which was recently established as an antiviral protein in humans (Mun and Punga 2019). Glycophorins (*GYPB* and *GYPA*), which determine MN and Ss blood groups in humans (Blumenfeld and Huang 1995) and define resistance to parasites, including malaria (Band et al. 2015), demonstrated high *F*_ST_ between the Yakut cattle and the pure taurine cattle with the *GYPB* being on top of the *F*_ST_ gene list (supplementary information 6, Supplementary Material online) and the positive selection list in Yakut cattle from the d*N*/d*S* analysis (MAF ≤ 0.1; supplementary information 9a, Supplementary Material online). Our finding of a convergent *NRAP* amino acid change in the Yakut cattle suggests that the Yakut cattle could be capable of slowing metabolism during the coldest winter periods. This was supported by the GO *metabolic process* (*q*-value < 0.02; supplementary information 10a, Supplementary Material online) and *integration of energy metabolism* (*q*-value = 0.04; supplementary information 10c, Supplementary Material online) Reactome terms enrichment in the HapFLK Yakut gene set. This adaptation would require changes to the heart’s ability to pump blood efficiently despite cold environmental temperatures. Indeed, the *cardiac conduction* Reactome pathway was overrepresented (*q*-value = 4.94e-05; supplementary information 11c, Supplementary Material online, and fig. 8) in the d*N*/d*S* gene set supported by the DAVID overrepresented cluster (enrichment score 2.00; supplementary information 12b, Supplementary Material online) combining terms *cardiac muscle contraction*, *dilated cardiomyopathy*, *hypertrophic cardiomyopathy*, etc. when the Yakut cattle *F*_ST_ was calculated against the pure taurine breeds from the 1000 Bull Genome Project data set and the same cluster with enrichment score of 1.32 when compared with all breeds (supplementary information 12b, Supplementary Material online). These categories included a common gene *RYR2*, which is central to myocardial excitation contraction coupling (Milting et al. 2006).

An important organ contributing to thermogenesis is the brown adipose tissue (Townsend and Tseng 2012; Fenzl and Kiefer 2014). In the Yakut cattle, one of the strongest HapFLK signatures of selection was in the area of the pleiotrophin (*PTN; q*-value = 4.42e-10; supplementary information 8, Supplementary Material online), a key gene in preserving insulin sensitivity, driving adipose tissue lipid turnover and the regulator of energy metabolism and thermogenesis (Sevillano et al. 2019). In line with this observation, we found that the *regulation of insulin secretion* term was enriched (*q*-value = 0.04; supplementary information 11c, Supplementary Material online) in the Yakut d*N*/d*S* gene set (fig. 8). This was further supported by the HapFLK analysis highlighting the Reactome categories *regulation of insulin secretion* (*q*-value = 0.01) and *integration of energy metabolism* (*q*-value = 0.04; supplementary information 10c, Supplementary Material online). Intriguingly, we identified two out of three candidate genes for cold/diet adaptation in native Siberian human populations (*PLA2G2A* and *ANGPTL8*; Hallmark et al. 2019) involved in lipid metabolism in the Yakut cattle HapFLK set. Another mechanism of reaction to extreme cold temperatures in mammalian cells is the cytoskeletal stabilization and/or disassembly including microtubules and actin filaments. In hibernating mammals, this is required for a rapid cytoskeleton reorganization during return from torpor to euthermy (Dave et al. 2012). We found multiple cytoskeleton protein genes in selected intervals of the Yakut cattle genome, including highly enriched DAVID clusters of *spectrin/alpha-actinin*, etc. (enrichment score 6.22); *myosin*, *actin-binding*, *microfilament motor activity, actin filament binding*, etc. (enrichment score 5.14) in the d*N*/d*S* set (supplementary information 11b, Supplementary Material online). Genes with the highest selection signals included actin-binding *SPTBN5* and *MYO10*, both of which were highlighted by our high-frequency SNP analysis. The *SPTBN5* is among 12 genes under selection in multiple Arctic and Antarctic species (Yudin et al. 2017). Another related group of cytoskeleton-related proteins under selection in the Yakut cattle were *pleckstrin homology-like domain* proteins in the d*N*/d*S* (enrichment score 2.80; supplementary information 11b, Supplementary Material online) and HapFLK (enrichment score 1.63; supplementary information 10b, Supplementary Material online) analyses. These domains are found in proteins that link the cytoskeleton to the cell membrane (Lenoir et al. 2015). One of these genes, *FARP1*, shows the highest HapFLK signal in the Yakut cattle (*q*-value < 1.37e-13; supplementary information 8, Supplementary Material online). An important organ of thermoregulation is the brain. It was shown (Schwartz et al. 2013) that the extracellular component proteins (collagens) play role in axonal guidance, synaptogenesis, and were overexpressed in the cerebral cortex during deep hibernation of ground squirrel. A similar pattern was observed in hypothalamic transcriptome of Djungarian hamster during torpor (Cubuk et al. 2017). The Reactome analysis of the d*N*/d*S* gene set highlighted multiple extracellular matrix terms enriched in the Yakut cattle, including *elastic fiber formation* (enrichment 3.48; *q*-value = 0.01); *laminin interactions* (enrichment 7.80; *q*-value = 3.2e-06), *assembly of collagen fibrils and other multimeric structures* (enrichment 5.05; *q*-value = 0.0005), *collagen formation* (enrichment 3.60; *q*-value = 0.01), etc. (fig. 8; supplementary information 11c, Supplementary Material online).

*Gene enrichment for Yakut SNP set absent or in low frequency in the European taurine cattle.* To understand probable contribution of the alleles, absent from European taurines, we looked for GO enrichment in the 13,257 SNPs with high frequency in the Yakut cattle. The *actin cytoskeleton* (*q*-value < 0.02) category was overrepresented in the set of SNPs that were found at low frequency (≤10%) in the European taurines, whereas the *response to stimulus* and *metabolic process* categories were enriched (*q*-value = 0.04) in the set of SNPs absent in the European taurine breeds (supplementary information 13a, Supplementary Material online). We observed that SNPs absent in the European taurines but found in Bovinae species were enriched in the GO category “*phospholipase A2 activity*” (*q*-value < 0.02), the Reactome pathway *Acyl chain remodeling of PG* (enrichment score = 3.7; *q*-value = 0.05; supplementary information 13c, Supplementary Material online), and “*MHC class I peptide loading complex*” (*q*-value < 0.02). Interestingly, the genes containing Yakut-specific SNPs were enriched in *biological regulation* (*q*-value < 0.02; supplementary information 13a, Supplementary Material online).

## Discussion

Comparison of demographic histories of the Yakut, Kholmogory, and Holstein breeds suggests an early separation of the Yakut cattle from European taurines. The estimated separation time (4,900 years ago) agrees with Payne and Hodges (Payne and Hodges 1997) who concluded that domesticated cattle appeared 4,000–5,000 years ago in the Northern East Asia, Northern China, Korea, and Japan. As this is a postdomestication event with the domestication itself dated to approximately 8,500–10,000 years ago (Loftus et al. 1994; Larkin and Yudin 2016), our data support a single taurine cattle domestication leading to both the European and Asian taurines. Our results, however, point to a much smaller *Ne* of the ancestral Asian taurine population compared with a much larger *Ne* of the population leading to European taurine breeds. This could be due to known historical admixture of European cattle population with wild aurochs (Upadhyay et al. 2017) until the European cattle’s *N_e_* reduced approximately 2,500 years. Interestingly, there is drastic decrease in Yakut cattle *N_e_* ∼ 300 years ago, which matches the period of the Yakutian history when in 1620s they came in contact with the expanding Tsardom of Muscovy. There is evidence that the Yakutian population went down by approximately 70% between 1642 and 1682 due to smallpox and other introduced infectious diseases (Wood 1991). Our data suggest that the Yakut cattle population could have gone through a severe bottleneck at the same time corroborating the event in the human history.

A high fraction of SNPs with high frequency in the Yakut cattle, absent from the European taurine breeds, but present in the indicine cattle, could imply historical introgression of these alleles into the Yakut cattle. However, the fact that vast majority of these alleles were also present in different Bovinae species implies that these variants belong to the pool of ancestral *B. primigenius* alleles shared with other *Bos* species. Another possible scenario which could potentially explain high genetic diversity of Yakut cattle at these positions is an ancient introgression with the East Asian aurochs (*B. primigenius*) that lived in the East Asian region during the arrival of taurine cattle together with several other Bovinae species, including yak, banteng, and gaur (Decker et al. 2014; Medugorac et al. 2017; Chen et al. 2018). Kantanen et al. (2009) have indeed suggested the near-eastern origins of the ancestral population of the Yakut cattle breed by studying mtDNA and Y-chromosomal diversity. This scenario, however, cannot explain large fraction of SNPs with a high frequency in the Yakut cattle, indicine cattle, African taurines, and with a low frequency in European taurines only. The fact that nearly two-thirds of missense alleles absent from the European taurines match orthologous amino acids found in majority of other phylogenetically distinct animal species also implies that these variants represent the ancestral state. We hypothesize that these putatively ancestral variants could be a rich reservoir of genetic variation important for adaptation of Yakut to extreme cold environment. This is indeed supported by their enrichment in genes from *response to stimulus/pain* and *immune system* categories potentially involved in adaptation to harsh environment. The fact that the *response to stimulus* category is under selection in another Northern Russian breed, the Kholmogory, confirms similar mechanisms of adaptation to cold climates in cattle breeds. Recently, the GO category response to stimulus was also found to be enriched in the list of genes with large number of missense mutations in the Finnish and Yakut cattle breeds (Weldenegodguad et al. 2018). A large fraction of genes in selected regions in the Yakut cattle genome belong to the *actin-binding* category; the same category was found enriched in the selected regions of the Kholmogory cattle, suggesting that these genes could contribute to cold adaptation as it was shown previously (Dave et al. 2012; Yurchenko, Daetwyler, et al. 2018; Banerjee et al. 2020). An actin-binding gene *SPTBN5*, which had the largest number of high-frequency missense mutations in Yakut cattle (21) compared with other taurine breeds, was shown to be under positive selection in at least three Arctic and Antarctic mammals including the wooly mammoth (Yudin et al. 2017; Lin et al. 2019) and in Chinese indicine cattle breeds (Mei et al. 2018).

Although the actin genes themselves and genes interacting with actin could contribute to multiple biological processes related to adaptation to cold climates (Banerjee et al. 2020), the only high-frequency Yakut-cattle specific missense mutation found in the actin-binding gene *NRAP* provides a clue for the likely mechanism. The fact that this H100Q variant, found in every single resequenced Yakut cattle animal was absent from all approximately 3,800 cattle animals from the 1000 Bull Genome Project, suggests its Yakut cattle origin. Moreover, its absence from all Bovinae species used in our work and ancient aurochs, yak, bison samples from Siberia suggests its relatively recent appearance in the Yakut cattle dating back to maximum 5,000 years ago, after separation from the European taurine branch. However, as the Yakut people have migrated to Yakutia in the 13^th^ century and other Asian, including the Turano–Mongolian breeds (Hanwoo), lack this variant as well indicates it could form as late as approximately 800 years ago in Yakutia followed by a very fast frequency increase in the Yakut cattle. While absent in cattle breeds and phylogenetically close Bovinae species, the exact same amino acid change has formed independently at least six times in mammalian evolution, in species lowering their metabolism in response to cold, including hibernating bats and lemurs, cold adapted deep-diving species, like walrus and sea lions. Interestingly, a potential contribution of a highly evolutionary conserved NRAP protein (Utama et al. 2002) to climate adaptation is supported by findings in a range of species including fish and reptiles (Gagnaire et al. 2012; Alderman et al. 2019; Capraro et al. 2019). *NRAP* is expressed exclusively in the skeletal and cardiac muscles and is involved in myofibrillar assembly and force transmission in the heart (Truszkowska et al. 2017), suggesting that the mechanism in Yakut cattle and other cold-adapted species could be related to the function of the heart. Human population studies show that the H100Q mutation is present but very rare suggesting its disadvantage when living in a “normal environment.” Human and mice mutations in *NRAP* leading to dilated cardiomyopathy (Ehler et al. 2001), a heart muscle genetic disorder, causing enlargement of the left ventricle and issues with pumping blood out of the heart point to possible negative effects. These could also include such cardiac dysfunction as arrhythmia which is a common condition reported for deep-diving animals (Williams et al. 2015) and rodents during arousal from hibernation (Eagles et al. 1988). We hypothesize that the *NRAP* variant plays a crucial role in adaptation to the coldest environmental temperatures in the Yakut cattle and other species, which would require enhanced heart ability to pump blood. This is supported by strong enrichment for genes from the cardiac contraction pathway in the Yakut cattle selected regions.

Scans for selection signatures pointed to multiple candidate genes and pathways for climate adaptations in Russian cattle breeds. The Yakut cattle contain a large number of genes with signatures of negative selection and a very few with signatures of positive selection. A possible explanation could be that the ancestral variants present in the Yakut cattle genome already make them a good fit into their environmental conditions, therefore a very few novel functional changes (like one in the *NRAP* gene) are required. Alternatively, it is possible that some additional functional changes could affect either regulatory gene regions or be represented by a different type of mutations not covered in this work, for example, by indels. Genes with the highest d*N*/d*S* selection signals in the Yakut cattle included genes involved in the cytoskeleton stabilization which plays an important role in the hibernating mammals during return from torpor to euthermy (Dave et al. 2012), pointing to another possible common mechanism of reaction to extreme cold temperatures in the Yakut cattle and other animals. Genomic regions potentially involved in adaptation to extreme environment, highlighted in this study, might well be affected by other factors like the physiological and morphological status of the animal. In particular, the Yakut cattle exhibit unique morphoanatomical adaptations to the arctic climate, like small live weight, deep and narrow chest, and short, firm legs (Kantanen 2009).

In conclusion, this work has revealed different evolutionary histories of two Russian northernmost cattle breeds, of which one is a typical taurine cattle and another contains multiple ancestral genetic variants, which allowed its adaptation to harsh conditions of living above the Polar Circle. Surprisingly, very few truly novel high-frequency genetic variants were required for this extreme adaptation, but the only detected missense change of this type represents a unique example of a young amino acid residue convergent change limited to a single cattle breed but shared with at least 16 species of hibernating/cold adapted mammals from six phylogenetic orders.

## Materials and Methods

### Sample Selection and Genome Sequencing

To choose animals for whole-genome resequencing (20 Yakut and 20 Kholmogory), we utilized 26 Yakut and 39 Kholmogory DNA samples previously genotyped on a 150 K SNP array (Yurchenko, Daetwyler, et al. 2018). After the QC and linkage-disequilibrium pruning, we calculated pairwise PI_HAT measure (Proportion IBD, that is, *P*(IBD = 2) + 0.5*P(IBD = 1)) of relatedness using plink (–genome) software among all the individuals for each breed separately. The majority of animals of Kholmogory breed were unrelated and for Yakut were third-degree relatives or unrelated (supplementary fig. 3, Supplementary Material online). All DNA samples were obtained from our previous study (Yurchenko, Daetwyler, et al. 2018). Next-generation sequencing (NGS) paired end reads (PE, 150 bp) were generated using HiSeq sequencing platform at Novogene Co., Ltd., Hong Kong, China. Library preparation was carried out by Novogene, and raw sequence data per sample, ranging from 41.1 to 66.8 Gb, were generated. In addition, we obtained raw sequence data of 153 cattle individuals from the NCBI SRA database, including 20 Holstein (Daetwyler et al. 2014), 20 Hanwoo (Shin et al. 2014), 47 African cattle (Ankole—10, Boran—9, Kenana—9, N’Dama—10, Ogaden—9), 36 Chinese cattle (Luxi—5, Nanyang—5, Jiaxian Red—5, Dianzhong—6, Wannan—5, Ji’an—4, Leiqiong—3, Yanbian—3), 5 Brahman, 10 Yak (*B. grunniens*), and 15 of other *Bos* species (5 Gayal [*B. frontalis*], 5 Banteng [*B. javanicus*], 3 Gaur [*B. gaurus*], and 2 Bison [*Bison*; supplementary information 2, Supplementary Material online; Mei et al. 2018; Chen et al. 2018]). Reference Hereford cattle (*B. taurus*) assembly (UMD3.1, *BosTau6*) was downloaded from NCBI.

### Read Mapping and Variant Calling

All cleaned reads were mapped to the cattle reference assembly using BWA-MEM (Li and Durbin 2009) with default parameters. The average mapping rate of the reads generated in this study was 99.68%, and the average sequencing coverage was approximately 11.43× (ranging from 9.7 to 15.1, table 1). Alignment preprocessing steps and variant calling were done following the Genome Analysis Toolkit (GATK v. 3.8; DePristo et al. 2011) pipeline. For each raw BAM file, we marked duplicate reads with Picard (v. 1.69) using the tool MarkDuplicates (http://broadinstitute.github.io/picard/, last accessed March 24, 2021). Next, we performed base quality score recalibration (using cattle known variants: dbSNP148). We followed the best practice guidelines (DePristo et al. 2011) recommended for variant discovery and genotyping using GATK v.3.8 with default parameters. First, genotype likelihoods were calculated separately for each sequenced animal using HaplotypeCaller, which resulted in files in gVCF (genomic Variant Call Format) format for each sample. Subsequently, GenotypeGVCFs was applied to genotype polymorphic sequence variants for multiple samples simultaneously. In details, three joint VCF files were generated for: 1) the Yakut, Kholmogory, Holstein, and Hanwoo breeds, 2) the Yakut, Kholmogory, Holstein, Hanwoo, Indian, Chinese and African breeds, and 3) the Yakut, Kholmogory, Holstein, Hanwoo, Indian, Chinese and African breeds, and Bovinae species samples (see supplementary information 2, Supplementary Material online, for samples). The filtering of SNPs for quality (hard filtering) was applied with the following parameters: 1) variant confidence/quality by depth < 2, 2) RMS mapping quality (MQ) < 40.0, 3) Phred-scaled *P*-value using Fisher’s exact test to detect strand bias > 60, 4) *Z*-score from the Wilcoxon rank sum test of alternative vs. reference read MQs (MQRankSum) < −12.5, and 5) *Z*-score from the Wilcoxon rank sum test of alternative vs. reference read position bias (ReadPosRankSum) < −8. The thresholds for these parameters were adopted from GATK Best Practices (DePristo et al. 2011). Multi-allelic SNPs and INDELs were removed, and the final filtered VCF file was used for further analysis.

### Genomic Annotation of SNPs

Whole-genome hard filtered SNPs were annotated using the Ensembl Variant Effect Predictor (VEP) with Ensembl v. 94 (McLaren et al. 2016). SNPs were annotated with the classification categories of impact HIGH, LOW, MODERATE, and MODIFIER (supplementary information 1, Supplementary Material online). Moreover, dNdScv (Martincorena et al. 2017), used to estimate whole-genome d*N*/d*S*, also classified SNPs based on the RefSeq Release 103 (Pruitt et al. 2012).

We derived a subset of SNPs from the four breeds’ VCF files with the following characteristics: 1) high frequency of alternative (nonreference) allele in Yakut cattle (Yakut alternative allele frequency of ≥70% and ≤10% in Holstein, Kholmogory, and Hanwoo) and 2) SNPs present in at least 25% of the four-breed sample set. NGS annotation pipeline (Grant et al. 2011) was used to assign classification (e.g., intronic, missense, synonymous, splice variant, stop gain, etc.) to each SNP in this subset of SNPs and to provide several fields of information describing the affected transcript and protein, if applicable. NGS-SNP also reports NCBI, Ensembl, and UniProt IDs for genes, transcripts, and proteins when applicable. For missense SNPs, an “alignment score change” was calculated by comparing the reference amino acid and the nonreference amino acid to an orthologous amino acid in a range of genomes. A positive value indicated that the alternative allele amino acid was more common in the orthologous position in other species than the reference amino acid, whereas a negative value indicated that the reference amino acid was more common (supplementary information 14, Supplementary Material online).

### Demographic History Reconstruction

To calculate historical demography and separation time between pairs of populations, we used two complementary approaches: SMC++ (Terhorst et al. 2017) and GADMA (Noskova et al. 2020), which use different population inference models. To remove spurious variants and regions with bad mapping quality from further consideration, we first computed the mappability score of the bovine reference genome using the GEM mappability program (Derrien et al. 2012) with kmer size =100 bp allowing one mismatch. The regions with mappability < 1 were removed. Additionally, we removed all regions under selection identified in the bovine genome (Yurchenko, Daetwyler, et al. 2018) and sex chromosomes.

SMC++ uses a sequential Markovian Coalescent approach to infer demographic history of separate populations and pairwise time of divergence between them on the unphased samples. The drawback of this method is an inability to implement migration into the model. For the SMC++ we analyzed demographic history using the cubic spline approach with mutation rate per bp per generation = 1.2e-8.

The approach implemented in the GADMA software is based on the comparisons of observed and modeled joint allele frequency spectrums of populations (Gutenkunst et al. 2009; Jouganous et al. 2017) and allows the efficient modeling of demographic parameters and migration, given a divergence scenario with up to three populations, using a powerful genetic algorithm. We ran GADMA with five years as cattle generation time using ten processes simultaneously and linked loci. The main algorithm chosen was moments (ordinary differential equations).

### Detection of Genetic Introgression between Breeds

We applied a robust forward–backward algorithm implemented in RFMix (Maples et al. 2013) to screen for the presence of putative taurine or indicine haplotypes in autosomes of the Yakut cattle. This algorithm uses designated reference haplotypes to infer local ancestry in designated admixed haplotypes; thus, five genetic groups were selected as a reference panel: European taurine (Holstein), Russian taurine (Kholmogory), African indicine (Ogaden), Chinese indicine (Wannan, Ji’an, and Leiquiong), and Indian indicine (Brahman). Window size was set to three (-w 3) and the option “–reanalyze-reference” with three iterations was used to analyze the reference haplotypes as if they were query haplotypes (Maples et al. 2013).

We conducted TreeMix v. 1.12 (Pickrell and Pritchard 2012) analyses to infer the relationships, divergence, and major mixtures among 18 cattle breeds and five Bovinae species (supplementary information 1, Supplementary Material online). We applied the option “-root YAK,” which sets Yak as the position of the root, option “-k 1000,” which builds the tree using blocks of 1000 SNPs to account for linkage disequilibrium and used the option “-se” to calculate standard errors of migration proportions. We allowed up to 15 migration edges on the tree (m ranging from 0 to 15) and generated a residual heatmap to identify populations that were not well-modeled after adding each migration edge. The percentages of variation explained by the maximum likelihood trees were also calculated. Migration edges were considered until 99.9% of the variance in ancestry between populations was explained by the model. We also ensured that the incorporated migration edges were statistically significant. Residuals from the model were visualized using the *R* script implemented in TreeMix. Finally, in order to provide further support for a past admixture between populations, we calculated *f3* and *D* statistics using ADMIXTOOLS (v. 5.1) with default parameters (Patterson et al. 2012). We calculated *f3* statistics using qp3Pop of the form (X; A, B) where a negative value mean implies that X is admixed with populations close to A and B. We considered negative statistics with Z-score values below 2 as significant signals of admixture.

We computed *D* statistics using qpDstat. *D* statistics of the form D (A, B, X, Y) to test the null hypothesis of the unrooted tree topology ((A, B), (X, Y)) was used. A positive value indicates that either A and X, or B and Y share more drift than expected under the null hypothesis. We quote *D* statistics as the Z score computed using default block jackknife parameters.

### Genome Scan for Selection Signals

#### Identification of Signatures of Selection with hapFLK Statistics

We performed a genome scan for selective sweeps within the groups of phylogenetically closely related breeds: Yakut and Hanwoo and Kholmogory and Holstein using haplotype-based statistics hapFLK (Fariello et al. 2013). Due to the hapFLK model assuming that selection acts on shared ancestral SNP allele frequencies, we excluded rare SNPs with low minor allele frequencies (MAFs) from breed groups (MAF < 0.1). We also excluded poorly genotyped individuals (<95% of SNPs with genotypes), loci genotyped in <99% of samples, SNPs without chromosomal assignments, and SNPs on sex chromosomes in PLINK, using the commands: –maf 0.1, –mind 0.05, –geno 0.01, and –chr 1-29 prior to performing the genome selection scans. This resulted in 8,423,043 and 7,926,039 SNPs for Yakut–Hanwoo and Kholmogory–Holstein groups, respectively.

The hapFLK method takes the haplotype structure of the population into account. What was important for our data set is that this method can account for population bottlenecks and migration. Reynolds distances and a kinship matrix were calculated by the hapFLK program v. 1.4 (Fariello et al. 2013). For the hapFLK analysis, the number of haplotype clusters for each breed group were estimated with fastPhase (Scheet and Stephens 2006) and were set as *-K* 10 for the Yakut–Hanwoo, and *-K* 20 for Kholmogory–Holstein groups. The expected maximum number of iterations was set to 30 for both groups. We applied midpoint rooting to all sets of breeds.

#### *P*-value Calculation

For hapFLK, the calculation of raw *P*-values was performed assuming that the selected regions represent only a small fraction of the genome (Fariello et al. 2014). The genome-wide distribution of hapFLK statistics could be modeled relatively well with a normal distribution except for a small fraction of outliers from potentially selected regions (Fariello et al. 2014). Robust estimations of the mean and variance of the hapFLK statistic were obtained using the *R* MASS package *rlm* function to eliminate influence of outlying regions following Biotard and coworkers (Boitard et al. 2016). This has been done for each group (Yakut–Hanwoo, and Kholmogory–Holstein). The hapFLK values were Z-transformed using these parameter estimates, and *P*-values were calculated from the normal distribution in *R*. The *R qvalue* package was used to correct *P*-values for multiple testing (Storey and Tibshirani 2003). To identify selected intervals, we scanned the hapFLK output for intervals containing *q*-values <0.01. The boundaries of the selected intervals were defined by the first marker up- and down-stream with a *q*-value >0.1. To assign a selection signal to a specific breed, Local Reynolds distances were calculated for selected regions using *local_reynolds*.*py* script and local population trees were then built with the *local_trees R* script obtained from https://forge-dga.jouy.inra.fr/projects/hapflk/wiki/LocalTrees (last accessed March 24, 2021).

#### Identification of Genomic Regions under Selection: dN/dS Ratio

Whole-genome d*N*/d*S* estimates were obtained for the SNPs found in any of the four cattle breeds (Yakut, Kholmogory, Holstein, and Hanwoo) using the dNdScv (Martincorena et al. 2017). The background mutation rate of each gene was estimated by combining local information (synonymous mutations in the gene) and global information (variation of the mutation rate across genes) and controlling for the sequence composition of the gene and mutational signatures. Unlike traditional implementations of d*N*/d*S*, dNdScv uses trinucleotide context-dependent substitution matrices to avoid common mutation biases affecting d*N*/d*S* (Greenman et al. 2006). The calculations were initially made using all hard-filtered SNPs (MAF in each breed ≥ 0.1) using the *Btau6* reference genome database generated and the “dndscv” function. The second run was done using hard filtered SNPs with MAF ≥0.6 in a breed to identify genes under selection which likely affected the whole breed. One-sided *P*-values were calculated and adjusted for multiple testing using Benjamini and Hochberg’s false discovery rate to detect both positive and negative selection (*q*-value positive selection and *q*-value negative selection).

### F_ST_ Calculations against the 1000 Bull Genome Data Set

Fixation index of genetic differentiation (*F*_ST_) between biallelic SNPs identified in our Yakut or Kholmogory cattle samples as part of their analysis by the 1000 Bull Genome Project Run 7 against the reference cattle ARS-USD1.2 genome was calculated against: 1) the set of pure taurine breeds (2,003 animals, 18 breeds) as identified from the structure analysis reported by Chen et al. (2018) and 2) against all cattle samples of taurine or indicine origins (3,362 animals) from the 1000 Bull Genome Project Run 7. This was performed using *vcftools* (Danecek et al. 2011) for each cattle autosome applying the following settings: *–fst-window-size 10000 –fst-window-step 5000*. All animals from the 1000 Bull Genome Project Run 7 have been filtered for a minimum of 6× mean genome coverage. One percent of the windows with the maximum mean *F*_ST_ values were extracted and overlapped with the gene set from Ensembl v. 94.

### Functional Enrichment Analysis and Protein Modeling

Cattle genes based on the Ensembl v. 87 database were retrieved using the Ensembl BioMart online tool (Smedley et al. 2009), and gene ontology (GO) analysis was performed using “indicine-like” genes from the RFMix analysis, and the hapFLK selected intervals using an in-house pipeline. Moreover, a DAVID functional cluster analysis (Huang et al. 2009) and a Reactome (Fabregat et al. 2017) pathway enrichment analysis were performed.

Genomic association test (GAT) (Heger et al. 2013) was used to estimate the significance of overlap between multiple sets of genomic intervals by using 10 K permutation. The genomic territory was represented by the *BosTau6* whole genome, and the tool was used to assess the significance indicine-like assigned genes from the RFMix analysis.

Protein sequences for several species and their alignments were retrieved from the UniProt database (Anon 2019). ESPript v. 3.0 (Robert and Gouet 2014) was used to gather sequence similarities and secondary structure information from aligned sequences. Next, Phyre2 (Kelley et al. 2015) allowed to predict protein 3D structure and estimate mutation sensitivity; the “intense” modeling option was used.

### Ancient and Historical Sample Analyses

Twenty-two bone specimens of ancient representatives of the genus *Bos* and *Bison* from the archaeological sites of Western and Eastern Siberia (Paleolithic—Early Iron Age) were investigated for the presence of G > T mutation at position chr26: 34,131,393. In addition, DNA from two sculls of the Yakut cattle (ID_290; dated 1926) and the Siberian cattle (ID_289; pre 1917) provided by the Museum of Livestock (Moscow Agricultural Academy) were investigated for mutation at the same position. All ancient bone specimens were subjected to UV irradiation; 1 cm^2^ of bone was crushed into powder in a metal mortar. Ancient DNA extraction was carried as described previously (Druzhkova et al. 2013) and historical DNA following the procedures described in (Abdelmanova et al. 2020). Primers for the fragment with the G > T SNP in the *NRAP* gene were selected using Primer3Plus v. 2.4.2 (Untergasser et al. 2012). А 72-bp fragment of the sequence was obtained using PCR in a volume of 30 μl, containing 20–50 ng of extracted ancient DNA, 1× Phusion HF Buffer (Thermo Fisher Scientific), 0.2 mM each of dNTP, 0.5 μM each of primer (NRAPF- TGTGGTTGCCTGTTAGGTG, NRAPR- ACGGTTTAAGTCGGTTTTTC), 400 ng/μl BSA (Bovine Serum Albumin), 1 e.a. of Phusion High-Fidelity DNA Polymerase (Thermo Fisher Scientific). Libraries of ancient DNA amplicons were obtained using TruSeq Nano DNA Sample Preparation Kit (Illumina) according to the manufacturer's High Sample Protocol with modifications. Pair-end sequencing of the libraries was performed on MiSeq platform (Illumina) using MiSeq Reagent Kit v2 (300 cycles). Read mapping to reference sequence (NM_001098072) was carried out in the program Geneious v. 10.0.5 (https://www.geneious.com, last accessed March 24, 2021).

## Supplementary Material

Supplementary data are available at *Molecular Biology and Evolution* online.

## Supplementary Material

msab078_Supplementary_Data
