# Supplementary material for: Demographic History, Adaptation, and NRAP Convergent Evolution at Amino Acid Residue 100 in the World Northernmost Cattle from Siberia
Source: Mol Biol Evol. 2021 Mar 30;38(8):3093–110. doi: 10.1093/molbev/msab078 (PMC8321547; doi:10.1093/molbev/msab078)
Supplement: msab078_Supplementary_Data [file msab078_supplementary_data.zip › Buggiotti et al_Supplemetary_Figure.pdf]

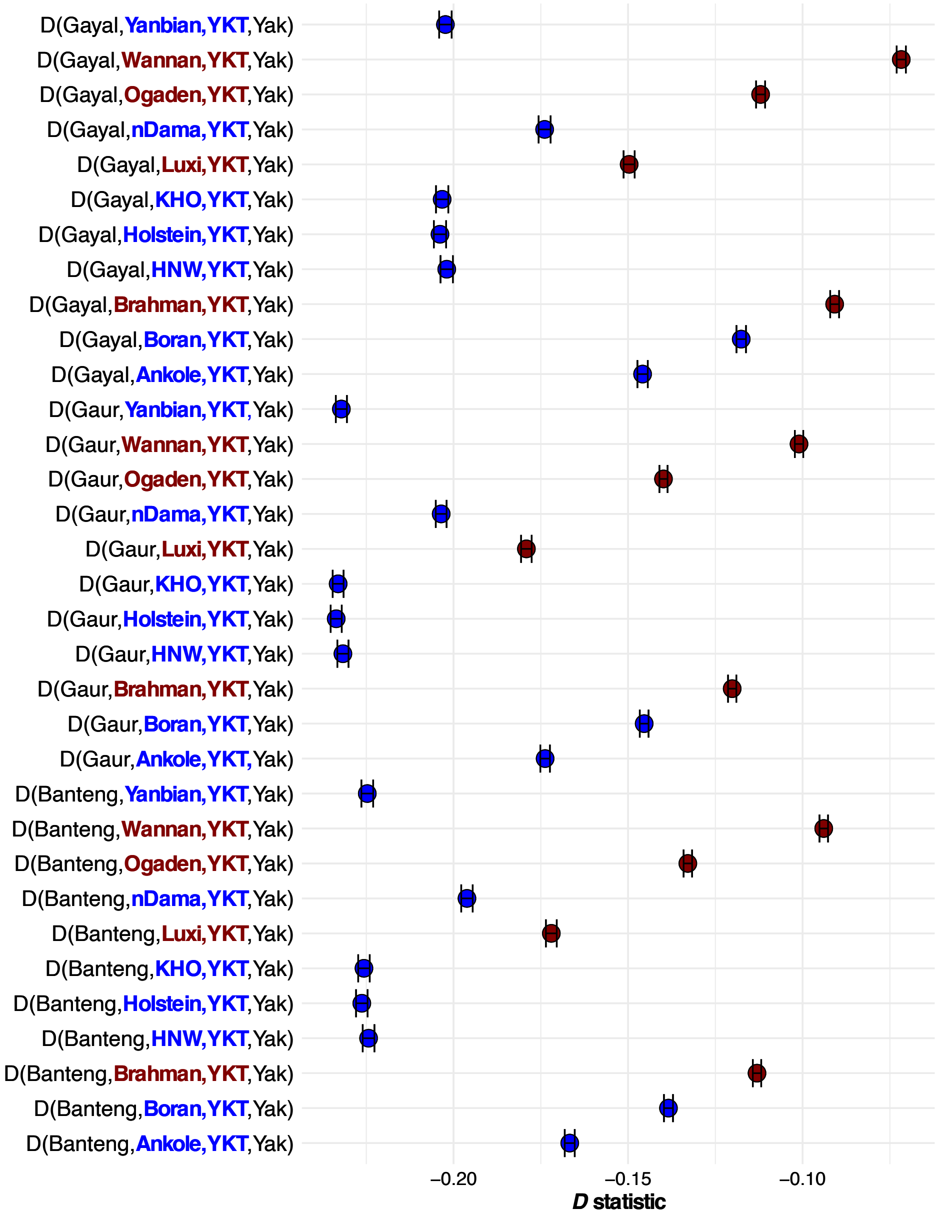

Supplementary Figure 1. D-statistics test, of the form D (A, B, X, Y), for admixture providing information about the direction of gene flow. Negative D-statistic indicates that gene flow occurred either between A and B or X and Y; cattle taurine breeds are in blue and cattle indicine breeds are in red. Abbreviations are as follow: YKT (Yakut), KHO (Kholmogory), HNW (Hanwoo).

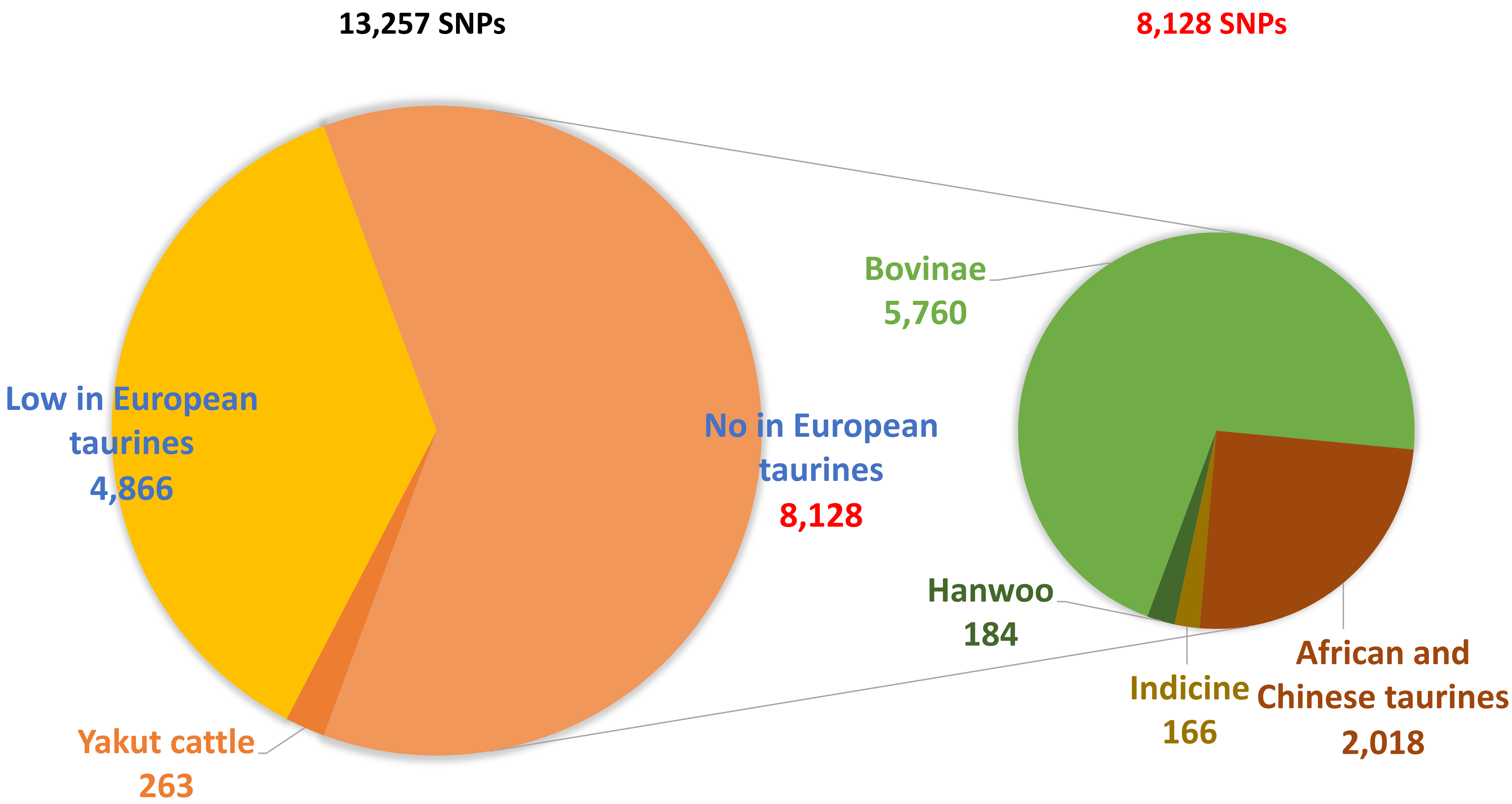

Supplementary Figure 2. Distribution of Yakut high frequency SNPs (total of 13,257 SNP) in European taurine, indicine cattle, Hanwoo, African, Indian, Chinese taurine, and bovinae species.

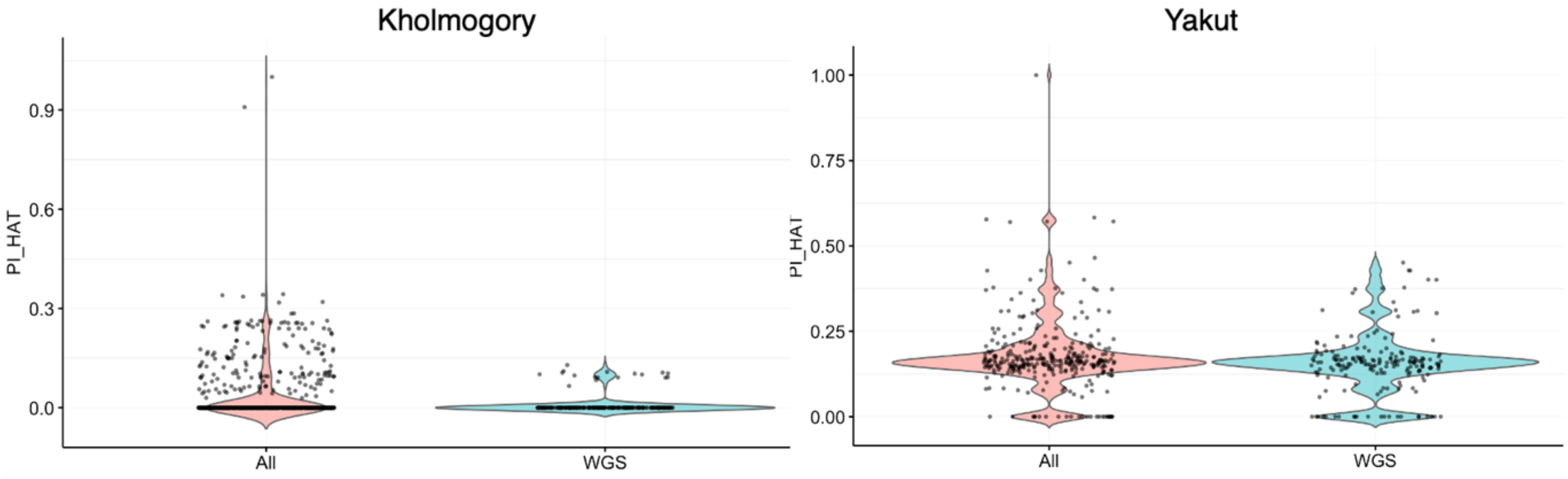

Supplementary Figure 3. Violin plot demonstrating the pairwise relatedness between animals chosen for genotyping (All; Yurchenko et al., 2018) and whole genome resequencing (WGS) analyses. PI_HAT measure can be interpreted like this: identical twins, duplicates = 1.0; first-degree relatives = 0.5; second-degree relatives = 0.25; third-degree relatives = 0.125. We choose the animals which have the lowest pairwise relatedness to others.
